# Perceptions and behaviours surrounding cannabis use during pregnancy, breastfeeding, and early parenting in Canada: a qualitative study

**DOI:** 10.1186/s42238-026-00469-x

**Published:** 2026-07-23

**Authors:** Justine Renard, Llisa Morrow, Jaismin Kaur Harneja, Laura G. Rosen

**Affiliations:** 1 Canadian Centre On Substance Use and Addiction, 75 Albert St, Suite 500, Ottawa, ON K1P 5E7 Canada; 2 Leger Marketing Inc, 507 Place d’Armes, Suite 700, Montreal, QC H2Y 2W8 Canada; 3 King’s University College at Western University Canada, 266 Epworth Ave, London, ON N6A 2M3 Canada

**Keywords:** Substance use, Cannabis, Pregnancy, Postpartum, Breastfeeding, Stigma

## Abstract

**Background:**

Cannabis use during pregnancy, breastfeeding and early parenting raises important public health concerns due to potential impacts on fetal and child development as well as parental health. However, individuals who use cannabis during these periods remain underrepresented in research. This study examines perceptions, behaviours, and decision-making related to cannabis use during pregnancy, breastfeeding, and early parenthood in Canada.

**Methods:**

A qualitative descriptive study was conducted using in-depth, semi-structured interviews. Thirty-one women aged 18–55 years were recruited across Canada. Eligible participants had used cannabis at least once in the past 10 years and reported experience with pregnancy, breastfeeding, or caring for a child under three years of age. Participants represented five Canadian regions and included English- and French-speaking individuals. Interviews were conducted online between April and May 2025, audio-recorded, transcribed verbatim, and analyzed using thematic analysis.

**Results:**

Analysis identified key themes related to: motivations for cannabis use, product types and modes of consumption, perceived health effects and benefits, interactions with healthcare providers, knowledge gaps, information sources, and experiences of stigma. Participants described having to weigh known benefits of cannabis against uncertain risks to unborn or young children in the context of receiving limited and inconsistent clinical guidance. Challenges disclosing cannabis use to healthcare providers contributed to seeking information from informal or non-clinical sources of information.

**Conclusions:**

This study addresses critical gaps in understanding cannabis use during pregnancy, breastfeeding, and early parenting in Canada. Findings highlight the need for clear, evidence-informed, and non-judgmental public health messaging and clinical communication to support informed decision-making and harm reduction among those who are pregnant, breastfeeding, and parenting young children.

**Supplementary Information:**

The online version contains supplementary material available at https://doi.org/10.1186/s42238-026-00469-x.

## Background

In Canada, cannabis was legalized for non-medical purposes in 2018. Since then, cannabis use has become more socially acceptable than prior to legalization (Health Canada 2024). While most Canadians recognize that there are harms associated with consuming cannabis, the prevalence of past-year cannabis use is increasing (Health Canada 2024). Along with growing acceptance and consumption comes concerns that groups who are at a greater risk of experiencing cannabis-related harms may also be increasing their use, such as young people and people with a history of psychosis (Fischer et al. 2022).

Cannabis use during pregnancy and breastfeeding has also emerged as an important area of research and public health interest. Recent data indicate that the prevalence of cannabis use among pregnant people in Canada has more than doubled from 1.2% in 2012 to 4.3% in 2022 (Tremblay et al. 2025). Within Ontario, pregnancies involving hospitalizations related to cannabis use increased by 82% (Myran et al. 2023) following legalization. While evidence continues to emerge, cannabis use during pregnancy has been associated with a range of adverse fetal and child health outcomes including: lower birth weight, preterm delivery, and increased likelihood of neonatal intensive care unit admission (Myran et al. 2023; Avalos et al. 2024; Baía and Domingues 2024; Corsi et al. 2019; Lo et al. 2024). Evidence also suggests possible associations between cannabis use in pregnancy and longer-term neurodevelopmental outcomes, including impacts on cognitive, emotional, and behavioral functioning in childhood (Corsi et al. 2020; Renard and Konefal 2022). The biological plausibility of these associations is supported by evidence that cannabinoids readily cross the placenta and may interact with the developing fetal endocannabinoid system, which is present by approximately five weeks of gestation, and is involved in key neurodevelopmental processes, including neuronal migration, synapse formation, and overall brain organization (Anavi-Goffer and Mulder 2009; Huizink and Mulder 2006; Wu et al. 2011).

While much of the literature has focused on cannabis exposure in pregnancy, the potential implications of cannabis use for infant and child health and development also extend into the breastfeeding and early parenting periods. Cannabinoids including Δ⁹-tetrahydrocannabinol (THC) can transfer into human milk and may remain detectable for extended periods because of their lipophilic properties (Baker et al. 2018; Moss et al. 2021). Although studies have demonstrated measurable cannabinoid concentrations in breast milk, evidence regarding potential effects on infant growth, neurodevelopment, and health outcomes remains limited and inconclusive (Renard and Konefal 2022; Castro-Navarro et al. 2024; Graves et al. 2022; Ordean and Kim 2020). Consequently, current clinical guidance generally recommends avoiding cannabis use while breastfeeding due to the potential for infant exposure and persistent uncertainties regarding possible harms (Graves et al. 2022; Ordean and Kim 2020).

As a stigmatized behaviour, the literature on motives, prevalence, and factors associated with cannabis use in pregnancy is emerging slowly. Cannabis use in pregnancy is reported to be motivated by managing physical symptoms of pregnancy such as nausea and for coping with psychological symptoms such as stress (Vanstone et al. 2021). Not completing high school, smoking or vaping tobacco, lower socioeconomic status, and having a partner that consumed cannabis all increase the odds of using cannabis in pregnancy (Kaarid et al. 2021; Manning and Drover 2020; Wilson et al. 2022). The literature on cannabis knowledge among pregnant and breastfeeding people is also limited. While many pregnant people believe that cannabis can be transmitted to babies in utero and through breastmilk (Bartlett et al. 2020), evidence suggests anywhere between 9–22% of pregnant people believe that cannabis use poses no risk to unborn or nursing babies (Manning and Drover 2020; Wilson et al. 2022). Concerningly, those who perceive themselves to have greater knowledge of the effects of cannabis in pregnancy are more likely to view cannabis as posing no harm to unborn babies (Wilson et al. 2022; Gould et al. 2025). This belief may partially result from the tendency for pregnant people to seek information about cannabis in pregnancy online before consulting a healthcare provider (Manning and Drover 2020; Bartlett et al. 2020; Bhatia et al. 2024; Gould et al. 2024). Further, increasing social acceptance and normalization of cannabis following legalization may also contribute to perceptions that cannabis use during pregnancy and breastfeeding is relatively safe or low risk (Manning and Drover 2020; Wilson et al. 2022; Bartlett et al. 2020; Chang et al. 2019; Denson et al. 2025).

The postpartum and early parenting periods are also important contexts in which cannabis use may occur. These periods are often characterized by increased caregiving demands, sleep disruption, and mental health challenges (Mental Health Research Canada 2025; Nelson et al. 2014) and may contribute to continued or resumed cannabis use among some individuals for reasons such as stress management, anxiety, and sleep difficulties. Emerging evidence suggests that parental cannabis use may influence parenting practices, parent–child interactions, and child wellbeing, although findings remain limited and inconsistent (Hill et al. 2018; Wesemann et al. 2022; Wilson and Rhee 2022). In addition, the presence of cannabis in households with infants and young children has become an increasing public health concern, with studies reporting substantial increases in unintentional pediatric cannabis poisonings since legalization (Myran et al. 2022, 2023).

Despite growing evidence regarding the potential effects of cannabis use during pregnancy, breastfeeding, and early parenting, important knowledge gaps remain regarding the lived experiences, motivations, information needs, and decision-making processes of individuals navigating cannabis use during pregnancy, breastfeeding, and early parenting. These gaps have been highlighted by healthcare providers, community partners, and individuals with lived and living experience, as well as through the Legislative Review of the Cannabis Act, which identified pregnant and breastfeeding individuals as priority populations for cannabis research and public health action (Government of Canada 2024). A better understanding of these experiences is needed to inform future research, public education initiatives, clinical guidance, and policy responses. To address these gaps, this study explored the perceptions, motivations, decision-making processes, and lived experiences of individuals in Canada regarding cannabis use during pregnancy, breastfeeding, and early parenting.

## Methods

### Study design

This study used a qualitative descriptive design based on in-depth, semi-structured interviews to examine individuals’ perceptions, behaviours, and decision-making related to cannabis use during pregnancy, breastfeeding, and early parenthood. This approach allowed for an in-depth understanding of sensitive and context-specific experiences, emphasizing participants’ own language and perspectives. The study adhered to the Consolidated Criteria for Reporting Qualitative Research (COREQ).

### Participants and recruitment

Participants were recruited through Leger’s LEO (Leger Opinion) online panel, which includes established participant validation procedures. A total of 3,399 individuals completed the screening survey, of whom 548 met the eligibility criteria. Among eligible participants, 145 expressed interest in participating in a follow-up interview and provided contact information. Of these, 73 were contacted, 42 were scheduled for an interview, and 31 completed an interview. Recruitment sought variation across cannabis-use experiences, age groups, and geographic regions; however, the sample was not intended to be statistically representative.

Participants in this study self-identified as women. Accordingly, gendered language is used in parts of the manuscript when describing participant experiences and quotations. However, the authors acknowledge that not all individuals who experience pregnancy, breastfeeding/chestfeeding, or parenting identify as women. Recruitment materials and screening procedures used inclusive language and did not include gender-based eligibility restrictions.

Participants were eligible to participate if they had used cannabis within the past 10 years and reported that their cannabis use, reduction, or cessation occurred during pregnancy, breastfeeding, or while caring for a child under three years of age. Parents of children aged three years and younger were included to capture experiences during the early parenting period, when caregiving responsibilities are intensive and issues related to child safety, supervision, and cannabis use may be particularly relevant.

Participants were recruited from five regions across Canada (Ontario, Quebec, Atlantic Canada, the Prairies, and Western Canada) via a web-based screening survey. Eligible participants were contacted by email, and eligibility was reconfirmed at the start of each interview.

Recruitment and analysis occurred concurrently, and continued until thematic saturation was reached, defined as the point at which no substantially new themes, concepts, or patterns emerged from successive interviews. Saturation was assessed through iterative review of interview transcripts, coding summaries, and regular discussions among members of the research team throughout the analytic process.

### Data collection

Data were collected between 1 April and 8 May 2025 through online interviews conducted individually via Microsoft Teams. Participants were invited to use video but could participate by audio only if preferred. Prior to each interview, informed consent and eligibility criteria were reconfirmed. The live, synchronous interview format allowed researchers to interact directly with participants in real time and clarify responses as needed.

Each session lasted approximately 30–60 min. Interviews were conducted primarily in English, and two sessions were held in French. No non-participants were present during interviews.

L.M. and J.H., qualitative researchers with formal training and professional expertise in qualitative research methods, conducted interviews from a non-clinical research perspective. Both interviewers were women. No prior relationship was established between interviewers and participants. Participants were informed that the interviews were being conducted on behalf of the Canadian Centre on Substance Use and Addiction to better understand experiences related to cannabis use during pregnancy, breastfeeding, and early parenting. Given the potentially sensitive and stigmatized nature of the topic, interviewers adopted a neutral, non-judgmental approach and emphasized confidentiality, voluntary participation, and that there were no right or wrong answers.

The semi-structured discussion guide (Supplementary Information) was collaboratively developed by the research team, whose expertise included cannabis research, public health, substance use, and qualitative methods. Development of the guide was informed by a scoping review of the literature (unpublished) and identified evidence gaps related to cannabis use during pregnancy and breastfeeding. Prior to data collection, the guide was reviewed and refined by the research team to improve clarity, flow, and relevance. The guide covered eight thematic domains: reasons for cannabis use, product types and consumption methods, cannabis access and the impact of legalization, perceived health effects and benefits, interactions with healthcare providers, perceived knowledge, information sources and needs, stigma, and social perceptions.

All interviews were audio-recorded and transcribed verbatim. Twenty-nine interviews were completed with both audio and video enabled. Two interviews were conducted using audio only due to technical difficulties with participants' cameras. No formal field notes were recorded beyond the interview transcripts. Participants received a CAD $175 honorarium in recognition of their time and contribution**,** including participation in a 30- to 60-min interview on a potentially sensitive topic involving cannabis use during pregnancy, breastfeeding, and early parenting. The honorarium was intended to provide fair compensation for participants' time and lived experience rather than to influence participation. Participation was voluntary, and participants could decline to answer questions or withdraw from the study at any time without penalty.

### Data analysis

Data were analyzed using an inductive thematic analysis informed by Braun and Clarke's approach to thematic analysis (Braun and Clarke 2022). L.M. and J.H. reviewed transcripts and summary notes to become familiar with the data and independently identify recurring ideas. Initial codes were developed inductively from the interview data and refined iteratively as additional transcripts were reviewed. Structured transcript review and coding matrices were applied for analysis; no specialized software was used. Related codes were grouped into higher-order categories which were subsequently organized into descriptive and interpretive themes reflecting patterns across participants’ experiences. Candidate themes were reviewed and refined against the full dataset to ensure they accurately reflected both shared patterns and variation across pregnancy, breastfeeding and early parenting contexts. Differences in interpretation were resolved through discussion and, where needed, review of the original transcript excerpts.

To ensure credibility and rigor, the research team used analyst triangulation, iterative review, and reflexive discussion throughout the analytic process. Researchers reflected on how their professional backgrounds, assumptions, and perspectives might influence analysis and interpretation, and regularly discussed these considerations as themes were developed and refined. Regular review of transcripts and supporting quotations helped ensure that themes remained closely aligned with participants' experiences and perspectives. Transcripts were cross-checked against recruitment profiles to verify group classification. Analytic decisions were documented systematically to maintain transparency. Transcripts were not returned to participants for comment.

### Ethics approval

The study protocol was reviewed and approved by the King’s University College Research Ethics Review Committee. All participants provided informed verbal consent prior to their interview.

## Results

### Participant characteristics

Thirty-one women aged 18–55 years representing five regions across Canada participated in this study. Participants were categorized into four analytic groups based on their experiences with cannabis use during pregnancy, breastfeeding and early parenting (see Supplementary Information for detailed demographic characteristics):

1. Cannabis use during pregnancy (*n* = 15), including participants who maintained pre-pregnancy use frequency (*n* = 3) or reduced use during pregnancy (*n* = 12).
2. Cannabis use while breastfeeding (*n* = 19), including participants who maintained (*n* = 2) or reduced (*n* = 17) use compared with pre-pregnancy frequency.
3. Stopped cannabis use during pregnancy and breastfeeding (*n* = 10), including participants who abstained throughout both periods.
4. Cannabis use while caring for a child under three years of age (*n* = 7)

Several participants contributed to more than one analytic group; therefore, the total number of group assignments exceeded the total number of participants.

### Patterns and motivations for cannabis use

Across all participant groups, cannabis use was described as purposeful and therapeutic rather than recreational. Participants who continued to use cannabis during pregnancy, breastfeeding, or early parenting most commonly indicated psychological (anxiety, mood regulation, and sleep difficulties) and physical (pain, migraines, nausea, pregnancy-related symptoms, and chronic health conditions) motivations for their use. No participants reported initiating cannabis use for the first time during pregnancy or breastfeeding. Several participants positioned cannabis as a preferable alternative to alcohol or prescription medications, particularly when seeking to manage anxiety, emotional reactivity, nausea or sleep.

“I had a lot of pelvic and hip pain during pregnancy…If I smoked a joint, it would help me a lot.”– Consumed during pregnancy (maintained use), 34, AB

*“I had really bad morning sickness and nothing was helping…The midwife prescribed me something for nausea. The only thing that helped me have an appetite and keep food down was cannabis.”- Consumed during pregnancy (reduced use), 25, BC.*

### Risk perception and mitigation

Participants consistently described a strong sense of responsibility toward their child, which influenced how they perceived cannabis use. Participants emphasized a thoughtful, individualized process of weighing perceived benefits against potential risks, with decisions evolving across pregnancy, breastfeeding, and early parenting. For those using cannabis as an established therapeutic tool (as discussed above), cannabis was often considered preferable to pharmacological alternatives due to perceived safety and fewer side effects. Stopping cannabis entirely was perceived as carrying its own risks, including unmanaged symptoms, increased anxiety, or reduced functioning. Some participants in this group described actively managing perceived risks by using lower doses than before pregnancy and avoiding high-THC products to prevent unwanted effects such as “brain fog.”

*“I thought that [cannabis] was a better option. Obviously, I wouldn’t want to drink in pregnancy…So that was the lesser of the evils…My anxiety…I found it helps to even it out.”- Consumed during pregnancy and breastfeeding (reduced use), 45, ON.*

Participants who reduced their use during pregnancy or breastfeeding continued to recognize cannabis’s therapeutic value but expressed heightened concern about potential harms. Uncertainty about fetal or infant exposure, combined with perceived lack of guidance from healthcare providers, led these participants to modify their use rather than discontinue it entirely. Concerns in this group included potential THC transfer through breast milk, reductions in milk supply, infant lethargy or irritability, and possible developmental outcomes such as delayed speech or motor milestones. These participants described adjusting their use with the goal of minimizing perceived risk, including limiting frequency of use, and modifying timing around breastfeeding. Practices such as spacing use from feeding sessions or discarding breastmilk (“pump and dump”) after use were commonly described.

“I thought about it [risks], yes. But I weighed the costs and benefits, and the amount I was using.”– Consumed during pregnancy and breastfeeding (reduced use), 31, MB

Participants who stopped using cannabis typically did so as a precautionary measure, often upon learning they were pregnant. Decisions to stop were driven by concerns about fetal or neurodevelopmental harm such as low birth weight, miscarriage, physical abnormalities, and long-term cognitive delays. Stopping was generally described as easier for those who had used cannabis recreationally than for those who had relied on it for symptom management, for whom cessation involved weighing personal well-being against potential risks to the child. Several participants compared cannabis to alcohol when weighing risks and described abstinence as the safest choice in the absence of clear evidence.

“I view cannabis in a similar way to alcohol or cigarettes—something to avoid during pregnancy or breastfeeding.”– Stopped during pregnancy and breastfeeding, 37, ON

### Product types and methods of use

Before pregnancy, most participants reported smoking cannabis in the form of joints, primarily due to its rapid onset and perceived ease of dose control. During pregnancy, breastfeeding, and early parenting, many participants reduced or avoided smoking because of concerns related to smoke exposure, inhalation, odour, and visibility. As a result, participants commonly adapted both the products and the ways they used cannabis.

“I didn’t like the smoke...I occasionally would try edibles and things that were less [intense].” - Consumed during breastfeeding (reduced use), 38, SK

Across groups, vape pens and ingestible oils were frequently preferred for their discretion, quick effects, and ease of use. Some participants also used edibles such as gummies or baked goods, though delayed onset and variable dosing were often described as difficult to manage. Product potency was also an important consideration across the sample. Lower-potency products were generally preferred. However, a small number of participants, typically those with longer histories of cannabis use, reported using higher-THC products in controlled contexts, most often at night to support sleep. Most participants preferred cannabidiol (CBD)-dominant products to manage anxiety, pain, or sleep while avoiding the psychoactive effects of THC. Topical products were mentioned occasionally for localized pain relief but were used infrequently.

*“I like to relax mostly, so I like to have a higher dose of CBD.” - Consumed while breastfeeding (reduced use), 34, QC*

Participants who maintained use during pregnancy or breastfeeding reported greater familiarity with cannabis and confidence in managing dosage. In this group, inhalation of dried cannabis flower remained common, alongside CBD/THC oils, tinctures, and vape pens. A small number also reported using capsules or higher-THC edibles for managing chronic symptoms or sleep.

### Perceived knowledge and confidence in decision-making

Participants consistently described uncertainty regarding the safety and health effects of cannabis use during pregnancy and breastfeeding. Decisions to reduce, maintain, or stop use were more often informed by perceived risks and knowledge rather than by clear evidence.

Those who reduced use described awareness of gaps in both their own knowledge and the broader evidence base. In contrast, participants who maintained use reported greater confidence in their understanding of the risks of cannabis use, largely informed by prior experience. Many viewed cannabis as a safer alternative to pharmaceuticals and emphasized responsible use, including attention to dosage, strain, effects, and method of consumption, rather than abstinence. At the same time, they acknowledged that clear, evidence-based guidance on safe use in these contexts remains limited. Participants who stopped use reported the least confidence in their knowledge. Their decisions to quit were driven by concerns of fetal or infant exposure through the placenta or breastmilk, and resultant developmental, cognitive, or physical effects. In the absence of clear information, abstinence was viewed as the safest option*.*

*“…that uncertainty made me not want to take a chance. I didn’t want the baby to have some kind of illness or deformity because I did something that I probably shouldn’t have.”– Stopped during pregnancy and breastfeeding, 34, QC.*

### Legalization, product safety, and information sources

Legalization of recreational cannabis was widely perceived as a sign to warrant increased confidence in the safety of cannabis products, particularly through regulation, testing, and clearer labelling. While legalization did not substantially change access for most participants, it provided reassurance about contaminants and potency, which was especially important during pregnancy and breastfeeding.

“I just feel more at ease knowing that legal cannabis has to meet strict regulations and quality standards.”– Consumed while breastfeeding, 26, NB

Despite greater confidence in regulated products, participants across all groups reported limited access to clear, evidence-based information about cannabis use during pregnancy, breastfeeding, and early parenting. Cannabis was described as less frequently addressed in prenatal and breastfeeding education compared to alcohol and nicotine. Interactions with healthcare providers were frequently characterized as brief, discouraging, or lacking practical guidance, contributing to uncertainty and selective non-disclosure driven by fear of judgment.

“You’re told all the things you shouldn’t do but rarely offered guidance on what you can do to support your health.”– Consumed with a child under three, 35, B.C.

As a result, many participants relied on personal experience and informal information sources, including online searches (e.g., Google, WebMD, government health websites, often described as vague), social media platforms (Facebook, TikTok, Reddit), peers, and dispensary staff.

“I did a google search… ‘Can you smoke weed while breastfeeding or while pregnant’?” – Consumed during pregnancy and breastfeeding (reduced use), 34, AB

### Stigma, disclosure, and social norms

Stigma surrounding cannabis use was widely reported and influenced both disclosure and decision-making. Many participants anticipated judgment from healthcare providers, family members, and the broader public, particularly in relation to social norms surrounding early parenting. These expectations led to selective disclosure or non-disclosure of cannabis use.

*“They [Doctors] ask you: ‘Do you drink? Do you smoke? Do you do drugs?’ And I was just answering ‘No’ to everything…Because you don’t want to be judged.” – Consumed during pregnancy and breastfeeding (reduced use), 48, AB.*

Concerns about being perceived as irresponsible or as a “bad” parent influenced how participants managed their cannabis use and how openly they discussed it. Participants frequently noted that, despite legalization, cannabis remained more socially stigmatized than alcohol in the context of pregnancy and parenting. Some participants internalized this stigma, while others, particularly those using cannabis for medical or therapeutic reasons, were more likely to normalize or defend their use. In the case of the latter, disclosure was often limited to trusted individuals.

*“People will call them [parents] careless. Like, ‘Oh, you don’t care about your baby.’”– Consumed with a child under three, 26, AB*

*“The level of judgment women face during pregnancy or breastfeeding is already intense—you’re constantly being scrutinized…when you add cannabis use into that equation, even if it’s legal, it’s often not received well.”– Stopped while pregnant and breastfeeding, 35, B.C.*

### Cannabis use in the context of parenting young children

Among participants who used cannabis while caring for children under three years of age, decision-making emphasized boundaries rather than abstinence. Participants described setting clear limits around when and where use occurred and framed their decisions in terms of maintaining their capacity to parent safely and effectively. Use was commonly restricted to times when children were asleep or when another caregiver was present.

“Once I stopped breastfeeding and my child began growing older, I resumed cannabis use—always outside and well away from him.”– Consumed with a child under three, 35, BC

Cannabis was described as a tool for managing the physical and emotional demands of parenting, including stress, sleep difficulties, and emotional regulation. Many participants viewed cannabis as a form of self-care and, for some, as a substitute for alcohol. Use was perceived as compatible with responsible parenting and as supporting the ability to remain present and engaged with their children.

“It's good for me to have something to wind down…I feel like it's more natural instead of having a glass of wine or two.”– Consumed with a child under three, 30, AB

Discretion and functionality were important considerations in this group, using in ways that allowed them to remain alert and responsive while caregiving, such as consuming low-THC products. Several participants described modifying their method of consumption, such as switching from smoking joints to vaping or consuming edibles. Negative effects were infrequently reported.

## Discussion

This study provides an in-depth qualitative examination of cannabis use across pregnancy, breastfeeding, and early parenting in Canada, highlighting how individuals navigate use amid uncertainty, limited clinical guidance, and persistent stigma. Cannabis use was rarely described as recreational; instead, decisions to continue, reduce, or stop use reflected deliberate efforts to manage health needs while minimizing perceived risks to the fetus or child.

Consistent with previous qualitative research, participants in this study reported using cannabis to manage mental health symptoms, pain, sleep difficulties, and the physical and emotional demands of pregnancy and postpartum (Vanstone et al. 2021; Chang et al. 2019; Barbosa-Leiker et al. 2020; Bayrampour et al. 2019; Garner et al. 2022). Many participants described cannabis as a natural or safer alternative to prescription medications, particularly when medication options were limited or perceived as riskier during pregnancy or breastfeeding, echoing other findings in both pregnant and postpartum populations (Chang et al. 2019; Denson et al. 2025); Barbosa-Leiker et al. 2020; Bayrampour et al. 2019; Cameron et al. 2022; Jarlenski et al. 2016; Kiel et al. 2023). For these individuals, stopping cannabis entirely was viewed as carrying its own health risks, including unmanaged anxiety, pain, or reduced functioning. Cannabis use was typically intentional and self-regulated, aimed at maintaining emotional stability or managing chronic conditions. Together, these findings suggest that cannabis use in pregnancy and breastfeeding is often motivated by efforts to manage physical and mental health symptoms rather than recreational reasons alone. These decisions were further influenced by perceptions that cannabis may represent a safer or more natural alternative to pharmaceutical medications. The findings therefore point to broader challenges related to symptom management during pregnancy and breastfeeding, where some individuals perceive few acceptable or effective alternatives to cannabis. As such, public health and clinical approaches may benefit from acknowledging the underlying reasons for cannabis use and providing individuals with evidence-informed information about symptom management options, treatment alternatives, and potential cannabis-related risks. Notably, no participants reported initiating cannabis use during pregnancy or breastfeeding, suggesting that these periods do not represent a point of new uptake but rather a time where existing use is reassessed, modified, or discontinued.

Participants demonstrated a strong sense of responsibility toward their children, with decisions about cannabis use influenced by ongoing risk–benefit assessments rather than indifference to potential harm, consistent with previous studies (Barbosa-Leiker et al. 2020; Weisbeck et al. 2021). Participants differed in how they interpreted and responded to uncertainty, reflecting distinct trajectories of cannabis use. Those who reduced use adopted a harm-reduction approach, adjusting dose, frequency, timing, or product type to limit perceived exposure while retaining therapeutic benefits. In contrast, participants who stopped use typically described abstinence as a precautionary choice in the absence of clear evidence, often drawing parallels between cannabis and alcohol use during pregnancy. In general, participants who maintained use expressed greater confidence in their decisions, often grounded in personal experience and symptom control, while still acknowledging gaps in the evidence base. Importantly, participants’ harm reduction-oriented behaviors, such as avoiding high-THC products or modifying patterns of use, were rarely informed by clinical guidance. These findings align with earlier work suggesting that in the absence of clear messaging from healthcare providers, decisions regarding cannabis use in pregnancy and breastfeeding are often informed by personal experience and perceived risks and benefits (Denson et al. 2025; Barbosa-Leiker et al. 2020; Weisbeck et al. 2021). More broadly, the findings suggest that individuals navigated uncertainty in different ways, with some adopting precautionary abstinence and others developing harm reduction strategies that they believed would minimize potential harms while preserving therapeutic benefits. Together, these findings illustrate how, in the absence of definitive evidence or practical guidance, individuals are often left to interpret and manage risks on their own.

A central finding of this study is that participants perceived a lack of accessible, evidence-based information on cannabis use during pregnancy, breastfeeding, and early parenting. Participants consistently described insufficient guidance from healthcare providers, with discussions often limited to general recommendations to stop use, without practical information about risks, alternatives, or harm reduction. This contributed to cannabis use non-disclosure due to fear of judgment, while those who did disclose often described clinicians as uncertain, dismissive, or inconsistent in their advice. This aligns with previous research from both Canada and the United States indicating that healthcare providers often feel unprepared to counsel patients on cannabis use during pregnancy or breastfeeding due to uncertainty in the evidence base (Bhatia et al. 2024; Jarlenski et al. 2016; Young-Wolff et al. 2020; Crowley et al. 2022; Panday et al. 2022). Without clear or explicit guidance, patients may interpret this lack of direction as an indication that cannabis use poses little or no risk (Bayrampour et al. 2019). The findings highlight an important need for improved education, training, and resources to support healthcare providers in discussing cannabis use during pregnancy, breastfeeding, and early parenting. Participants were often seeking practical information about risks, alternatives, and ways to reduce potential harms, rather than recommendations to abstain alone. As such, clinical approaches may benefit from moving beyond abstinence-focused messaging to include patient-centered discussions that acknowledge uncertainty, address individual circumstances, and provide evidence-informed information on symptom management, treatment alternatives, and potential risk-reduction strategies. Such conversations may help build trust, encourage disclosure, and create opportunities for ongoing support, education, and safer decision-making. Consequently, in the absence of clear, practical, and evidence-informed guidance from healthcare providers, many participants turned to alternative sources of information to support their decision-making. Consistent with prior qualitative research (Barbosa-Leiker et al. 2020; Kiel et al. 2023; Taneja et al. 2023), participants in this study reported turning to peers, social media, or cannabis retailers for advice, despite seeking more credible information to guide their decision-making (Denson et al. 2025). Although these sources offered practical support and a sense of community, participants also acknowledged their inconsistency and limitations, demonstrating the persistent information gap faced by individuals navigating cannabis use during pregnancy, breastfeeding, and early parenthood. For instance, after using cannabis some participants discarded expressed milk to minimize perceived infant exposure to cannabinoids, but evidence suggests that this strategy provides a false sense of protection (Moss et al. 2021; Wymore et al. 2021). This illustrates that although individuals are actively attempting to reduce risks, they lack access to accurate, evidence-based information to guide safer practices. Ultimately, this highlights a gap between clinical recommendations and the realities of decision-making among individuals who use cannabis during pregnancy and breastfeeding.

Legalization of non-medical cannabis did not materially change cannabis access for most participants, but it did increase confidence in product safety through regulation, testing, and labelling; factors that were particularly important for many participants. For some parents, legalization also contributed to normalization of cannabis as a means of relaxation or self-care, occasionally positioning it as an alternative to alcohol. Following legalization, stigma surrounding cannabis use during pregnancy and early parenting remained significant. Participants described anticipating judgment from healthcare providers, family members, and society more broadly, particularly in relation to expectations of “good motherhood.” Cannabis was perceived as more stigmatized than alcohol in parenting contexts, reinforcing selective disclosure and internalized guilt. These findings echo broader literature documenting the moralization of substance use during pregnancy and the disproportionate scrutiny placed on mothers (Stone et al. 2015; Weber et al. 2021). The persistence of stigma, despite legalization, suggests that legal acceptance of cannabis does not necessarily translate into social acceptance, particularly when cannabis use occurs during pregnancy or early parenting. Social norms and expectations surrounding pregnancy, parenting, and caregiving continue to strongly influence how cannabis use is perceived, discussed, and managed, even within a legalized context. These findings highlight the enduring influence of stigma on individuals' experiences and highlight the need for continued public education and professional training to address misconceptions, reduce stigma, and support more informed and non-judgmental discussions about cannabis use during these life stages.

Altogether, these findings have important implications for clinical practice, public health, research, and policy. Participants described making decisions regarding cannabis use in the context of symptom management needs, uncertainty, and limited evidence regarding potential risks and benefits. The findings demonstrate the importance of supporting individuals who are navigating these decisions while acknowledging the complexity and uncertainty that characterize the available evidence. They also highlight the need to equip healthcare providers with the knowledge, skills, and resources needed to support meaningful, evidence-informed, and non-judgmental conversations about cannabis use during pregnancy, breastfeeding, and early parenting. Finally, future research should prioritize addressing the evidence gaps most relevant to these life stages, including questions related to long-term effects, breastfeeding, parenting, product characteristics, dose, timing of use, and approaches to reducing potential harms.

### Strengths and limitations

This study has several limitations. First, as a qualitative investigation, the findings are not intended to be statistically generalizable to all pregnant, breastfeeding, or parenting individuals in Canada. Participants were recruited through an online panel, which may have resulted in a sample that differs from the broader population of pregnant and breastfeeding people and parents to young children. Individuals willing to participate in research on this topic may hold specific perspectives or levels of comfort discussing cannabis use, which could further influence the findings. Second, the study relied on self-reported data, which may be affected by recall bias or social desirability bias, particularly given the contentious nature of cannabis use in pregnancy, breastfeeding, and early parenthood. Although interviews were conducted confidentially, some participants may have underreported or reframed their experiences. In addition, interviews were conducted remotely via Microsoft Teams. While this approach facilitated participation from individuals across Canada and may have increased comfort discussing a potentially sensitive topic, it may also have influenced rapport and the depth of information shared during interviews. Third, while participants were recruited from multiple regions across Canada, certain populations may be underrepresented. Individuals from rural or remote communities, for example, may experience different challenges not fully captured in this study. Fourth, all study participants identified as women, so the experiences of transgender, nonbinary, and gender-diverse individuals are not reflected in this study. In addition, most participants identified as White, limiting the extent to which the findings capture the perspectives of racially and ethnically diverse populations. Finally, the inclusion criterion of cannabis use within the previous 10 years may have introduced variability in participants' experiences and recall. Participants' experiences spanned both pre- and post-legalization periods in Canada, which may have influenced factors such as access to cannabis, stigma, perceptions of risk, and product availability. Although the study was not designed to examine the impact of legalization, these contextual influences emerged in participant narratives and are reflected throughout the findings where relevant.

Despite these limitations, this study addresses critical gaps in Canadian public health research by examining cannabis use across pregnancy, breastfeeding, and early parenting, periods often studied independently of each other and rarely examined from the perspective of those directly affected. By including individuals who maintained, reduced, or stopped cannabis use, the study also captures a range of decision-making pathways relevant to public health prevention and harm reduction. The qualitative approach provided in-depth insight into how individuals navigate cannabis use in the context of limited clinical guidance and stigma. These findings offer valuable context for interpreting population-level trends and can inform public health messaging, provider education, and the development of non-judgmental, evidence-informed approaches to cannabis use in pregnancy, breastfeeding, and early parenthood in Canada.

## Conclusion

Cannabis use during pregnancy, breastfeeding, and early parenting is influenced by health needs, uncertainty about risks, limited clinical guidance, and persistent stigma. Participants described careful, context-dependent decision-making rather than casual use and expressed a clear need for non-judgmental, evidence-informed conversations that acknowledge uncertainty, address mental health needs, and support informed choices. Addressing persistent knowledge gaps and strengthening supportive dialogue in healthcare settings are critical to informed care among those who are pregnant, breastfeeding, or parenting young children.

## Supplementary Information

Supplementary Material 1.

## Data Availability

Available from the corresponding author upon reasonable request and subject to ethical approval.

## Abbreviations

COREQ: Consolidated Criteria for Reporting Qualitative Research
CBD: Cannabidiol
LEO: Leger Opinion
THC: Δ⁹-Tetrahydrocannabinol
