# Supplementary material for: Perceptions and behaviours surrounding cannabis use during pregnancy, breastfeeding, and early parenting in Canada: a qualitative study
Source: J Cannabis Res. 2026 Jul 23;8:111. doi: 10.1186/s42238-026-00469-x (PMC13688951; doi:10.1186/s42238-026-00469-x)
Supplement: Supplementary file 1 [file 42238_2026_469_MOESM1_ESM.docx]

APPENDIX A

Participant Demographics

*Table 1. Summary of Participants by Region, Age Group, and Cannabis Use Category*

| *Total n=31* | **Count** | **% of Total Sample** |
| --- | --- | --- |
| **Region** |  | |
| British Columbia | n = 3 | 10% |
| Alberta | n = 8 | 26% |
| Prairies (Manitoba & Saskatchewan) | n = 4 | 13% |
| Ontario | n = 11 | 35% |
| Québec | n = 3 | 10% |
| Atlantic Canada (New Brunswick) | n = 2 | 6% |
| **Age** |  | |
| 18-24 | n = 3 | 10% |
| 25-34 | n = 16 | 52% |
| 35-44 | n = 10 | 32% |
| 45-54 | n = 2 | 6% |
| **Cannabis Use Category** |  | |
| Group 1: During Pregnancy  *Reduced*  *Maintained* | **n = 15**  n = 12  n = 3 | **48%**  39%  10% |
| Group 2: While Breastfeeding/Chestfeeding  *Reduced*  *Maintained* | **n = 19**  n = 17  n = 2 | **61%**  55%  6% |
| Group 3: Stopped During Pregnancy and Breastfeeding/Chestfeeding | **n = 10** | **32%** |
| Group 4: With a Child Under 3 Years Old | **n =7** | **23%** |

*Table 2. Distribution of Cannabis Use Context by Region and Age Group*

|  | **Cannabis Use Category** | | | |
| --- | --- | --- | --- | --- |
|  | **Group 1: During Pregnancy**  (n=15) | **Group 2: While Breastfeeding/**  **Chestfeeding**  (n=19) | **Group 3: Stopped During Pregnancy and Breastfeeding/**  **Chestfeeding**  (n=10) | **Group 4: With a Child Under 3 Years Old**  (n=7) |
| **Region** |  | | | |
| British Columbia | 13% | 11% | 10% | 14% |
| Alberta | 33% | 32% | 20% | 43% |
| Prairies (Manitoba & Saskatchewan) | 13% | 11% | 10% | 0% |
| Ontario | 33% | 32% | 40% | 29% |
| Québec | 7% | 11% | 10% | 0% |
| Atlantic Canada (New Brunswick) | 0% | 5% | 10% | 14% |
| **Age** |  | | | |
| 18-24 | 20% | 11% | 0% | 0% |
| 25-34 | 53% | 58% | 50% | 57% |
| 35-44 | 13% | 21% | 50% | 43% |
| 45-54 | 13% | 11% | 0% | 0% |

****Note:*** *Nineteen participants qualified for multiple cannabis use categories across different stages (pregnancy, breastfeeding/chestfeeding, and with a child under three). As a result, group totals exceed 100%.*

APPENDIX B

Interview Guide

*Introduction and Rapport Building*

Thank you for joining me today. I’m here to learn about your experiences with cannabis use during pregnancy, chestfeeding and early parenting. There are no right or wrong answers, so feel free to share whatever you are comfortable with and there is any question you’re not comfortable answering, that’s fine too.

Your answers will be kept confidential, and your name will not be used in any report. Is it okay if I record this conversation to capture your feedback accurately? Know that the interview recordings will be securely stored for a maximum period of 6 months following the completion of the interview phase. After this time, the recordings will be permanently deleted using secure data erasure software to ensure complete removal. Access to the recordings will be restricted solely to the research team directly involved in the analysis, ensuring participant confidentiality and compliance with ethical research guidelines.

[OBTAIN VERBAL CONSENT]

**Do you have any questions before we begin? [CONFIRM GROUP(S)]**

To start, could you tell me a little about yourself? For example, what does a typical day look like for you?

*Questions*

**1. Cannabis Use Behaviours and Reasons**

*(Adapt questions based on the participant’s situation: pregnancy, chestfeeding, etc.*

- Can you tell me about your cannabis use experience during [pregnancy/ chestfeeding]? *[IF qualifying for multiple categories- ask for experience in each separately]*
- When did you start using cannabis? [PROBE: Was it before [pregnancy/ chestfeeding] and you continued use or did you start during [pregnancy/ chestfeeding]?]
- What were your reasons for using cannabis at that time? [HINT: managing nausea, stress, sleep, pain, recreational use].
- Did you continue using cannabis throughout [pregnancy/ chestfeeding]?
  - Have your reasons for using cannabis changed over time with change in circumstances? (e.g., from pregnancy to parenting)?

**[If stopped using cannabis during pregnancy or chestfeeding**]:

- What influenced that decision? [Probe: Was it something specific, like advice from a healthcare provider, personal concerns, or any experience?]
- How was the experience of making that change? Did it feel like a smooth transition, or were there any challenges along the way?
  - What challenges did you face? [ For example, cravings, stress, or find it hard to manage symptoms like nausea or anxiety without it]
- Looking back, is there anything that would have made it easier for you to stop using cannabis? For example, more information, resources, or support?

**2. Perceived Health Effects and Benefits**

- What effects did you notice from using cannabis during [pregnancy/ chestfeeding]? [IF qualifying for multiple categories- ask for effects in each separately]
  - Did it affect your physical health in any way? (e.g., nausea, sleep, appetite, pain, loss of weight)?
  - And, did it affect your mental health? (e.g., helped with or caused stress, anxiety, mood)?
- Did you experience any benefits from using cannabis during [pregnancy/ chestfeeding]?
  - Were there specific ways it helped you during [pregnancy/chestfeeding]?
- Did you experience any concerns or challenges related to your cannabis use during [pregnancy /chestfeeding]? [IF qualifying for multiple categories- ask separately for experience in each category]
- Were you worried about any risks to yourself, your baby, or your child?
  - Did you notice any negative effects?

**3. Types of Products and Methods of Use**

Now, I’d like to know a bit about the cannabis products that you have used during [pregnancy/ chestfeeding]:

- What types of cannabis products have you used?
  - Were you using the products that contained THC, CBD, or a mix of both? [PROBE: were there any reasons for using one over the other?]
  - Can you tell me more about the specific formats you’ve used? [For ex: smoking, vaping, edibles, oils, tinctures, topicals, etc.]
  - [IF qualifying for multiple categories] Did the type of product change during [pregnancy vs chestfeeding]? Why?
- Why did you choose these products and methods?
  - PROBE: Convenience, availability, personal preference, advice from others?
- When choosing products, did you think about factors like potency or dosage? [For example, did you look for something milder or stronger depending on your needs?]

**4. Cannabis Access and Legalization Impact**

- Where did/do you usually get your cannabis products?
  - [Probe: Legal store, unlicensed sources, friends, family, personal cultivation]
- Has legalization affected how you access cannabis or how you feel about using it?
- Did legalization affect your confidence in the safety or quality of the products you use(d)?
  - Did it make you feel more comfortable using cannabis during [pregnancy/ chestfeeding]?

**5. Perceived Knowledge of Cannabis**

- Based on what you know or experienced, how do you think, does cannabis affect people during [pregnancy/chestfeeding]? [IF qualifying for multiple categories- ask separately for each category]
  - Do you think it has specific benefits? If so, what are they?
  - Are there any negative effects or risks you are aware of?
- Is your knowledge about cannabis based on your own experiences, or did you learn about it from somewhere else?
  - [IF FROM SOMEWHERE ELSE: could you please share where you learned it from?
  - How reliable do you feel this source is, and why?

**6. Information Sources and Needs**

- Where do/did you get information about cannabis use during [pregnancy/ chestfeeding]?
  - PROBE: Healthcare providers, online sources, social media, friends, family, dispensaries, etc.?
  - Was it easy to find, or did you struggle to get the information you needed?
  - Was the information clear, helpful and reliable?
- Do you feel you had enough information to make decisions about cannabis use?
  - **If no:**
    - What information do you think is missing or unclear?
    - What specific questions or concerns did/do you have about cannabis use?
- What format would be most helpful for getting information about cannabis use? REALLY PROBE ON THIS.
  - Websites, brochures, videos, in-person discussions, support groups, Podcasts etc.? WHAT INFORMATION SHOULD BE INCLUDED, SPECIFICALLY.

**7. Interactions With Healthcare Providers**

- Did you talk to a healthcare provider (e.g., doctor, midwife, lactation consultant) about using cannabis during [pregnancy/chestfeeding]?
  - **If yes:**
    - What did they say, and how did their response make you feel?
    - Did you feel comfortable discussing cannabis with them? Why or why not?
    - Did you feel supported or judged?
    - Was the information they provided helpful?
  - **If no:**
    - What made you decide not to discuss cannabis use with your healthcare provider?
    - What would make you feel more confident or comfortable discussing cannabis with them?

**8. Stigma and Social Perceptions**

- How do others react to using cannabis during [pregnancy/ chestfeeding]? Overall, have you felt judged or supported?
  - Who did you feel this judgment or support came from (e.g., family, friends, healthcare providers)?
- Do you think there is stigma around using cannabis during pregnancy or chestfeeding?
  - Did this stigma affect your decisions or behaviour about cannabis use? How so?
  - Did you avoided discussing your cannabis use with others because of stigma?
- Do you ever think about how your cannabis use might be perceived by others, like your children, family, or friends?
  - Does this perception affect when, where, or how you use cannabis?
  - Do you think legalization has changed how people view cannabis use during [pregnancy/chestfeeding]?

*Closing Questions and Reflections*

- Is there anything we haven’t covered that you’d like to share about your experiences with cannabis use?
- What advice would you give to others in similar situations (pregnancy, chestfeeding)?
- What kind of support or resources do you think would be most helpful for people making decisions about cannabis use?

Thank you for your time and openness.
